# Management of a Novel Autoimmune Disease, COPA Syndrome, in Pregnancy

**DOI:** 10.1155/2022/4865985

**Published:** 2022-03-02

**Authors:** Archana Ayyar, Rachel D. Seaman, Kalpalatha Guntupalli, Mary C. Tolcher

**Affiliations:** ^1^Department of Obstetrics and Gynecology, Baylor College of Medicine, Houston, Texas, USA; ^2^Department of Medicine, Pulmonary and Critical Care, Baylor College of Medicine, Houston, Texas, USA

## Abstract

**Background:**

COPA syndrome is a rare autoimmune disease, demonstrating an autosomal dominant inheritance pattern with variable penetration that occurs more frequently in females than males. This disease manifests in childhood as pulmonary hemorrhage, arthritis, and renal disease.

**Case:**

We present a case of obstetric management of a 20-year-old nulligravida patient with a diagnosis of COPA syndrome. Her case was further complicated by multiple antepartum admissions for hypoxemia and a complex psychosocial history of substance use. On her first antepartum admission, rheumatology recommended management with hydroxychloroquine, inhaled corticosteroids (budesonide), and bronchodilators (albuterol inhaler) as needed. On admission for induction of labor, she was again noted to have oxygen desaturations. A chronic thrombus was noted on computed tomography (CT), and a multidisciplinary team was recommended against Valsalva. Thus, she had a primary cesarean delivery. Her postpartum course was only remarkable for improved oxygenation status.

**Conclusion:**

Management of COPA syndrome should be performed by a multidisciplinary team including maternal-fetal medicine, rheumatology, and pulmonology specialists. Traditionally, COPA syndrome is treated with immunomodulator therapy often used to treat autoimmune syndromes. However, many of these medications are not well studied or contraindicated in pregnancy. Preconception counseling is recommended both to ensure pregnancy safe medications being prescribed and to provide information on the genetic inheritance of this disease. At time of entry to care, patients should have a baseline work-up including a radiographic imaging, complete blood count, complete metabolic panel, lactate dehydrogenase, and a 24-hour urine protein collection for baseline. Although thought to be rare, COPA syndrome has an autosomal dominance pattern of inheritance with variable penetrance that is more common in females. Thus, incidence of COPA syndrome in pregnancy will likely increase in the future. Further case studies are warranted to optimize management of patients with COPA syndrome in pregnancy.

## 1. Introduction

COPA syndrome, named after its affected COP*α* protein, is a recently identified, heritable immunodeficiency marked by dysfunctional protein transport between the Golgi complex and endoplasmic reticulum [1–3]. Under physiologic circumstances, COP*α* regulates the retrograde movement of proteins from the Golgi complex to the endoplasmic reticulum. One of these proteins is the stimulator of interferon gene (STING), which provides immune system equilibrium. Dysfunctional transport by mutated COP*α* in COPA syndrome leads to trapping of STING within the Golgi which facilitates a skewed type I interferon inflammatory response [4, 5]. This leads to the release of proinflammatory cytokines and abnormal cellular autophagy, manifesting clinically as diffuse alveolar hemorrhage (DAH), arthritis, and renal disease—often beginning before five years of age and progressing into adulthood [1–3, 6].

First identified in 2015, COPA syndrome is thought to be more common than previously believed [3]. Following an autosomal dominant inheritance pattern, the syndrome appears to have variable penetrance and affects females more commonly than males. However, to our knowledge, this is the first documented case of COPA syndrome in pregnancy.

## 2. Case

A 20-year-old primigravida with known COPA syndrome presented at 11 weeks gestation to establish prenatal care at our maternal-fetal medicine clinic. Her COPA disease had been diagnosed at an early age, requiring multiple admissions for pulmonary vasculitis with alveolar hemorrhage throughout her childhood. Secondary to a complex psychosocial situation and financial instability, she had been off immunosuppressive therapy for two years at the time of her entry into prenatal care. During that period, she sought symptomatic relief of episodic dyspnea through local emergency departments, with her most recent encounter occurring at 6 weeks gestation. At that visit, she presented with mild shortness of breath and was discharged with a rescue albuterol inhaler. She also endorsed long-term, regular use of cigarettes and marijuana, and smoking cessation education was provided.

On presentation to our obstetrics clinic, the patient complained of persistent shortness of breath and was found to have a peripheral oxygen saturation of 88% upon ambulation. She was directly admitted to the antepartum service for further evaluation of her hypoxemia. Chest X-ray revealed diffuse reticular opacities consistent with a history of interstitial lung disease, without any evidence of active pulmonary hemorrhage. Pulmonary function testing demonstrated a mild restrictive ventilation defect [FEV1 2.26 L (71% of predicted value), FVC 2.57 L (72%), and FEV1/FVC 88% (101%)]. Echocardiogram was normal with an ejection fraction of 60-64% and without evidence of right-sided dysfunction or pulmonary hypertension. Supplemental oxygen was not needed throughout her work-up, and her oxygen saturation remained largely within normal limits for the remainder of the admission. Following consultation with rheumatology, she was discharged home on hydroxychloroquine 300 mg daily, budesonide twice daily, and albuterol inhaler as needed, with close outpatient follow-up. She was readmitted at 21 weeks gestation for similar symptoms, thought to be due to medication nonadherence. Work-up was again unremarkable, she did not require oxygen therapy, and she was discharged to home.

At 37 2/7 weeks gestation, she was admitted for induction of labor in the setting of newly diagnosed fetal growth restriction with an estimated fetal weight at the 3^rd^ percentile. However, upon presentation, she was again noted to be hypoxemic, with oxygen saturations ranging from 74 to 94% on room air. She required supplemental oxygenation via nasal cannula and her clinical picture was concerning for a COPA flare with possible diffuse alveolar hemorrhage (DAH). She again acknowledged nonadherence with her home medications and recent cigarette smoking at the time of admission.

An extensive work-up was commenced, and admission chest X-ray and echocardiogram remained stable from prior. Computed tomography of the chest revealed poor visualization of the superior vena cava (SVC) with extensive collateral vasculature of the left hemithorax and mediastinum, concerning for a chronic SVC thrombus with resultant collateralization and possible SVC syndrome. This was corroborated by review of outside hospital records which revealed a stable SVC thrombus for the past two years, thought to have arisen from a prior port placement. A multidisciplinary discussion was held amongst maternal-fetal medicine, cardiology, pulmonology, hematology, and radiology specialists, and ultimately, the decision was made to proceed with prednisone 30 mg daily for presumed COPA flare. Anticoagulation was deferred in the setting of suspected chronic thrombus, given the potential adverse effects of concurrent alveolar hemorrhage. The decision was also made to proceed with cesarean delivery for maternal benefit as Valsalva was felt to pose an indeterminable risk for dislodgement of the SVC thrombus and/or pulmonary decompensation.

Delivery itself was unremarkable. The patient underwent a primary low transverse cesarean delivery under neuraxial anesthesia and without cardiopulmonary complications. Her pulmonary status improved following delivery, and she was weaned off supplemental oxygen by the second postoperative day. Prophylactic anticoagulation was again deferred given remote risk for alveolar hemorrhage. The patient completed a three-day prednisone course and resumed her home medications. She was subsequently discharged on postoperative day three with close outpatient follow-up.

## 3. Discussion

COPA syndrome is a novel immunodeficiency due to mutations of the regulatory COP*α* protein and dysfunctional intracellular protein transport, resulting in interferon-mediated inflammatory response. COPA syndrome has a myriad of clinical manifestations, most commonly presenting as diffuse alveolar hemorrhage and antibody-mediated conditions (e.g., rheumatoid factor-positive arthritis, antineutrophil cytoplasmic antibody-positive arthritis and lung disease, isolated juvenile idiopathic arthritis, or isolated lupus-like nephropathy) [3].

Our index case was diagnosed with COPA syndrome in early childhood, and her course was complicated by discontinuity of care from diagnosis through adulthood, leading to numerous admissions for pulmonary vasculitis with alveolar hemorrhage. Her case was further complicated by numerous barriers to care leading to medication nonadherence and by marijuana and tobacco use—all of which likely exacerbated her chronic underlying pulmonary damage.

Treatment for COPA syndrome is similar to other immunodeficiency and autoimmune disorders and utilizes immunomodulators with varying safety profiles for use in pregnancy. In 2015, the Food and Drug Administration (FDA) replaced the previous pregnancy risk categories A-D and X with a new system entitled Pregnancy and Lactation Labeling Rule. This new system is aimed at providing data and clinical considerations for pregnancy, lactation, and reproduction that allow for clinical interpretation and patient-specific counseling [7]. Acute COPA exacerbations may be managed with systemic corticosteroids, cyclophosphamide, or rituximab. Maintenance therapy can include etanercept, intravenous immunoglobulin, azathioprine, hydroxychloroquine, or methotrexate [6–8]. While methotrexate is contraindicated during pregnancy, the other medications may be considered on an individual basis after appropriate discussion with specialists and patient counseling. The role of surgical management remains investigatory, although Mallea et al. report a case of a COPA patient undergoing lung transplantation with stable pulmonary function fifteen months postoperatively and without evidence of disease recurrence [9].

This case was largely managed via expert opinion, drawn from a diverse, multidisciplinary healthcare team. Although a multidisciplinary healthcare team may be involved in the care of these patients, we recommend that the obstetrical care of COPA patients be managed by maternal-fetal medicine specialists. Ideally, intensive preconception counseling should be performed in order to review patterns of transmission to offspring and to discuss potential risk of worsening maternal morbidity, particularly the risk of pulmonary decompensation. Radiographic imaging, echocardiography, and pulmonary function testing should be obtained at entry to prenatal care, serving as a baseline for comparison in event of an antepartum disease flare.

Without prior reports of COPA syndrome in pregnancy, it is difficult to discern the effects of pregnancy on COPA syndrome. However, one can infer that pregnancy increased the oxygen demands in this patient, as her previously required oxygen supplementation was weaned down postpartum. Furthermore, although there are no studies on COPA syndrome in pregnancy, there are reports of DAH secondary to systemic lupus erythematosus (SLE) [10]. Alveolar hemorrhage can cause severe hypoxemia that is unresponsive to mechanical ventilation [11]. In SLE patients, abrupt alveolar hemorrhage may develop despite concurrent immunosuppressive drugs. Treatment for active alveolar hemorrhage includes high-dose corticosteroids, possible addition of cyclophosphamide, ventilatory support, and blood resuscitation [10]. Although the patient in this index did not have acute DAH, the same treatment for DAH can be used as a model for future COPA syndrome patients with concurrent DAH.

As previously mentioned, COPA syndrome may also manifest as lupus-like nephritis, with the resultant proteinuria mimicking a common pregnancy condition, preeclampsia. Our index patient did not develop blood pressure elevation, so this conflict did not present itself. However, it is reasonable to also include preeclampsia labs (e.g., complete blood count, liver function testing, lactate dehydrogenase, creatinine, and urine protein studies) in the baseline evaluation of maternal COPA syndrome. This may aid in diagnostic clarity should the patient develop elevated blood pressures in later gestation.

Lastly, the complexities of delivery planning should not be underestimated in the setting of COPA syndrome. Mode of delivery for our index patient was determined based on multiple factors including her active COPA flare causing desaturation, chronic SVC thrombus, and possible SVC syndrome. On literature review, alveolar hemorrhage does not appear to be absolute contraindication to Valsalva; however, it is also important to note that there are no reports associating chronic SVC thrombus with COPA syndrome. Although our patient ultimately underwent a cesarean section, delivery planning for patients with COPA syndrome should be individualized without a clear contraindication to attempted vaginal delivery.

Given COPA syndrome's variable penetrance and increased expression in women, it is likely that the incidence of COPA syndrome in pregnancy will increase in the future. Further research is warranted to optimize the management of obstetrics patients with COPA syndrome.
